# Glucose levels between the anterior chamber of the eye and blood are correlated based on blood glucose dynamics

**DOI:** 10.1371/journal.pone.0256986

**Published:** 2021-09-01

**Authors:** Toshihide Kurihara, Deokho Lee, Ari Shinojima, Taku Kinoshita, Saori Nishizaki, Yoko Arita, Yuki Hidaka, Yasuyo Nishi, Yoshinori Shirakawa, Sho Kimura, Yukari Tsuneyoshi, Hidemasa Torii, Kazuo Tsubota, Kazuno Negishi

**Affiliations:** 1 Laboratory of Photobiology, Keio University School of Medicine, Tokyo, Japan; 2 Department of Ophthalmology, Keio University School of Medicine, Tokyo, Japan; 3 Fuji Xerox Co., Ltd, Tokyo, Japan; 4 SEED Co., Ltd., Tokyo, Japan; 5 Tsubota Laboratory, Inc., Tokyo, Japan; University of Florida, UNITED STATES

## Abstract

Glycemic control is essential to manage metabolic diseases such as diabetes. Frequent measurements of systemic glucose levels with prompt managements can prevent organ damages. The eye is a glucose highly demanding organ in our body, and the anterior chamber (AC) in the eye has been suggested for a noninvasive blood glucose monitoring site. However, calculating blood glucose levels from measuring glucose levels in AC has been difficult and unclear. In this study, we aimed to examine glucose levels from AC and find a correlation with blood glucose levels. A total of 30 patients with cataracts (men and women, study 1; 7 and 3, study 2; 9 and 11) who visited Keio University Hospital from 2015 to 2018 and agreed to participate in this study were recruited. Glucose levels from AC and the blood were examined by a UV-hexokinase or H_2_O_2_-electrode method before/during the cataract surgery. These values were analyzed with regression analyses depending on the groups (blood glucose-ascending and descending groups). In the blood glucose-descending group, glucose levels from AC were strongly correlated with blood glucose levels (a high R^2^ value, 0.8636). However, the relatively moderate correlation was seen in the blood glucose-ascending group (a low R^2^ value, 0.5228). Taken together, we showed different correlation ratios on glucose levels between AC and the blood, based on blood glucose dynamics. Stacking data regarding this issue would enable establishing noninvasive blood glucose monitoring from measuring glucose levels in AC more correctly, which will be helpful for proper and prompt managements for glucose-mediated complications.

## Introduction

Glycemic control is considered important for metabolic diseases such as diabetes mellitus [1]. The Diabetes Control and Complications Trials report indicated that tight control of blood glucose levels, through testing glucose levels frequently and adjusting insulin concentrations concomitantly, may prevent chronic diabetes complications [2]. Otherwise, uncontrolled blood glucose levels can result in chronic hyperglycemic stresses to blood vessels, finally causing severe complications such as diabetic retinopathy, diabetic nephropathy and diabetic neuropathy [3, 4].

Frequent testing of blood glucose levels can be performed by repetitive lancing or finger bleeding, termed “self-monitoring of blood glucose (SMBG)” [5]. SMBG is the most commonly used method all around the world [5]. When patients need to know their blood glucose levels, they puncture their hands (usually, fingers), put the blood sample to the testing gadgets, and blood glucose levels are immediately determined [5], which is very easy to use. However, their methods can cause fear of testing, pain and scarring after testing [5]. Additionally, they provide a potential door for bacterial or viral entries [5]. Repetitive punctures can damage chronic nerve fibers at the measurement sites [5], and production of biohazardous wastes after testing can be another issue for the environment. In consideration of these matters, a next generation of a testing method came to the surface on medical equipment fields, termed “continuous glucose monitoring (CGM) devices” [6]. CGM devices are mainly inserted in the abdomen, and their probe tubes are placed in the body [6]. The glucose concentration in tissue fluids can be measured by CGM devices, of which detection is not direct glucose levels in the blood [6]. With real-time monitoring, patients can get the data at any time and CGM devices have alarms to alert the patients for proper managements against sudden changes of glucose levels. However, setting up CGM devices in the body is still hurting [6]. Next, to solve several matters about existing testing methods, new monitoring devices have been developed, termed “flash glucose monitoring (FGM) devices” [7]. The way of measuring glucose levels is noninvasive in that the devices only use one flash shortly to sense the amount of glucose levels in interstitial fluids [7]. However, FGM devices could also induce skin irritation as they need to be attached on the skin for use. Taken together, even though there have been several developments for glucose level testing, noninvasive and effective methods are still highly on demand.

The eye is one of the highest energy (directly referred to as glucose) consuming organs in our body for ocular physiological functions [8]. Previous reports suggested that the aqueous humor in the anterior chamber of the eye could be used for measuring blood glucose levels noninvasively [9–11]. The aqueous humor is a glucose-containing body fluid secreted from non-pigmented epithelial cells of the ciliary body, which are connected via tight junctions, forming the blood-aqueous barrier [12]. Advantages of the aqueous humor for measuring glucose levels are its high accessibility as it is close to the surface, and the cornea is clear to see-through [9–11]. Furthermore, there has been a notion that its glucose levels may appear linearly dated to blood glucose levels based on the results from several animal and human studies [9, 13–15]. Thus, researchers have attempted to use the aqueous humor for measuring blood glucose levels noninvasively. However, there has been a discrepancy between glucose levels in the blood and the eye [16, 17], which implies dynamics of glucose levels in our body still need to be well understood.

In this study, we examined glucose levels in the aqueous humor from the anterior chamber of the eye and the blood, and attempted to find a relationship between them. Moreover, we propose a concept of glucose level dynamics in the anterior chamber and the blood, which will be useful to establish an infrastructure for future noninvasive ocular glucose monitoring systems in a correct way. This further will help the technical development of glucose managements for complications of diabetes including diabetic retinopathy.

## Methods

### Ethics statement and subject selection

This prospective study was approved by the Institutional Review Board of Keio University Hospital in 2014 (Study 1, approval no. 20140436, UMIN000017362) and in 2016 (Study 2, approval no. 20160217, UMIN000025885) and adhered to the tenets of the Declaration of Helsinki. In these studies, inclusion and exclusion criteria were applied to select the current study subjects. Among the subjects who visited the Department of Ophthalmology at Keio University Hospital, the subjects who (1) were older than 20 years old, (2) planned to have a cataract surgery, and (3) agreed to participate in this study (a written form of the consent document was provided to the subjects), were chosen to meet inclusion criteria. The subjects who had a history of diagnosis for severe complications other than cataracts were excluded. The subjects who have an extremely shallow anterior chamber (the depth of the anterior chamber: 2.5 mm or less) and who need legal representative consents, not themselves, for the agreement to take part in this study were also excluded. Finally, 10 subjects for Study 1 and 20 subjects for Study 2 were enrolled as our current research subjects.

Systemic diseases in selected patients for this study were as follows: Study 1; diabetes (n = 5), hypertension (n = 5), benign prostatic cancer and tuberculosis (n = 1), cardiac infarction (n = 1), heart disease (after aortic valve replacement) (n = 1), diabetic macular edema (after panretinal laser photocoagulation) (n = 1), Wolff-Parkinson-White syndrome (after operation) (n = 1), atrial fibrillation (n = 1), or constipation (n = 1). Study 2; diabetes (n = 4), hypertension (n = 3), both retinal tears (after laser photocoagulation) (n = 1), retinal detachment in the right eye (after operation) (n = 1), asthma (n = 1), cerebral aneurysm (n = 1), Hashimoto’s disease and an epiretinal membrane in the left eye (n = 1), chronic hepatitis C (n = 1), glaucoma (n = 2), atrial fibrillation (n = 1), valvular disease (n = 1), subdural hematoma (n = 1), aortic dissection (after operation) (n = 1), hyperlipidemia (n = 3), reflux esophagitis (n = 1), keroid (n = 1), benign prostatic hyperplasia (n = 3), or gout (n = 1). Basically, treatments and care of the subjects were included depending on the diseases.

### Cataract surgery

Cataract surgery was conducted using a Constellation system and/or an INFINITI Vision system (Alcon Laboratories Inc., Fort Worth, TX). Topical eye drop anesthesia (0.4% oxybuprocaine hydrochloride and 1% lidocaine) was applied to all patients during the surgery. After disinfection using povidone iodine and draping, corneoscleral or corneal main incisions were performed using a 2.2 or 2.4 mm width blade. One or two side points were created with a 1.0 mm blade approximately 90° on either side of the main incision. OPEGAN Hi (purified sodium hyaluronate, Santen Pharmaceutical Co., Ltd., Japan) and VISCOAT (purified sodium hyaluronate and chondroitin sulfate sodium, Alcon Japan Ltd., Japan) were used as ophthalmic viscoelastic devices (OVDs). Continuous curvilinear capsulorhexis was made using bent 27-gauge needles or Inamura capsulorhexis forceps. Hydrodissection was conducted using viscoelastic cannulas. After using the phaco chop technique and removing the cataract nucleus via phacoemulsification, cortical cleanup was conducted using an irrigation/aspiration (I/A) handpiece (or bimanual I/A handpiece depending on the cases). A capsular bag was expanded with cohesive OVDs followed by posterior chamber intraocular lens insertion. After aspiration of OVDs, anterior chamber pressure was confirmed by using a balanced salt solution, and stromal hydration was used to seal corneal incisions.

### Sample preparation and glucose level measurements

#### Study 1

Before the cataract surgery (the time that the patients entered the operation room; approximately less than 17 minutes before the surgery), blood samples were collected and measured by UV-hexokinase (ultraviolet absorption spectrophotometry, SRL Inc, Tokyo, Japan) and H_2_O_2_-electrode methods (Antsense Duo, Horiba, Tokyo, Japan). Approximately 2 mL of blood was collected in a blood collection tube containing NaF-EDTA/2Na (SRL Inc, Tokyo, Japan). Then, the tube was maintained at room temperature until analysis. The measurement of glucose levels was according to the protocols of the manufacturers. During the surgery, blood samples were collected (with the same method above) as possible to the time of collection of aqueous humor samples (S1 Fig). About 50 μL of aqueous humor samples were collected from a corneal incision during the surgery and the samples were diluted 10 times with distilled water for the measurement. After all, blood and aqueous samples were also measured by the two methods above.

#### Study 2

Before the cataract surgery (the time that the patients entered the operation room), blood samples were collected 2 times with a 5 mins interval (T1 and T2) (S2 Fig) and measured by the UV-hexokinase method. During the surgery, blood samples were collected 2 times with a 5 mins interval (T3 and T4). At the first blood sample collection (T3) during the surgery, the aqueous humor samples were also collected and measured by the same method above. The method for blood collection in Study 2 was the same with that in Study 1.

### Statistical analysis

All data analyses were performed with the R statistical software (R Core Team, Auckland, New Zealand) and the Prism 5 (GraphPad, San Diego, CA, USA). The single regression analysis statistical model was used for analyzing a correlation of glucose levels between the aqueous humor from the anterior chamber and blood. Coefficients (slope and intercept), F-statistic, R^2^ and 95% confidence intervals were estimated to show the strength of associations and p-values of less than 0.05 were considered as statistically significant. R^2^ was considered moderate with values of 0.4–0.69 and strong with values of 0.70–1.0 [18].

## Results

### Clinical characteristics of the study subjects

For Study 1, 10 subjects were adequately selected based on our criteria as described in Methods. Among them, 7 (70%) were men and 3 (30%) were women (Table 1). The average age of the subjects was 63 ± 16.1 years. For Study 2, 20 subjects were selected (Table 1). 9 (45%) were men and 11 (55%) were women. The average age of the subjects was 72 ± 9.3 years. Information on eye laterality, intraocular pressure, cataract nuclear grade, blood pressure, time of surgery, and SpO2 was also checked during the studies (Table 1).

**Table 1:** Clinical features of cataract patients.

Characteristics	All patients (n = 30)
Study 1	
Gender (man:woman)	7 (70): 3 (30)
Age (year)	63 (± 16.1)
Eye laterality (right/left)	6 (60): 4 (40)
Intraocular pressure (mmHg)	15.9 (± 3.3)
Cataract nuclear grades	2.1 (± 0.9)
(Emery-Little classification)	
Systolic blood pressure (mmHg)	135.0 (± 13.1)
Diastolic blood pressure (mmHg)	72.5 (± 12.4)
Time of surgery (min)	14.2 (± 8.9)
SpO2 (%)	97.7 (± 1.1)
Study 2	
Gender (man:woman)	9 (45): 11 (55)
Age (year)	72.0 (± 9.3)
Eye laterality (right/left)	10 (50): 10 (50)
Intraocular pressure (mmHg)	14.0 (± 2.0)
Cataract nuclear grades	2.0 (± 0.8)
(Emery-Little classification)	
Systolic blood pressure (mmHg)	139.0 (± 18.3)
Diastolic blood pressure (mmHg)	78.0 (± 9.9)
Time of surgery (min)	14.2 (± 8.9)
SpO2 (%)	98.0 (± 1.4)

Values are presented as average (± standard deviation) or number (%).

### Glucose levels from the anterior chamber and blood in the subjects

#### Study 1

**Fig 1:**
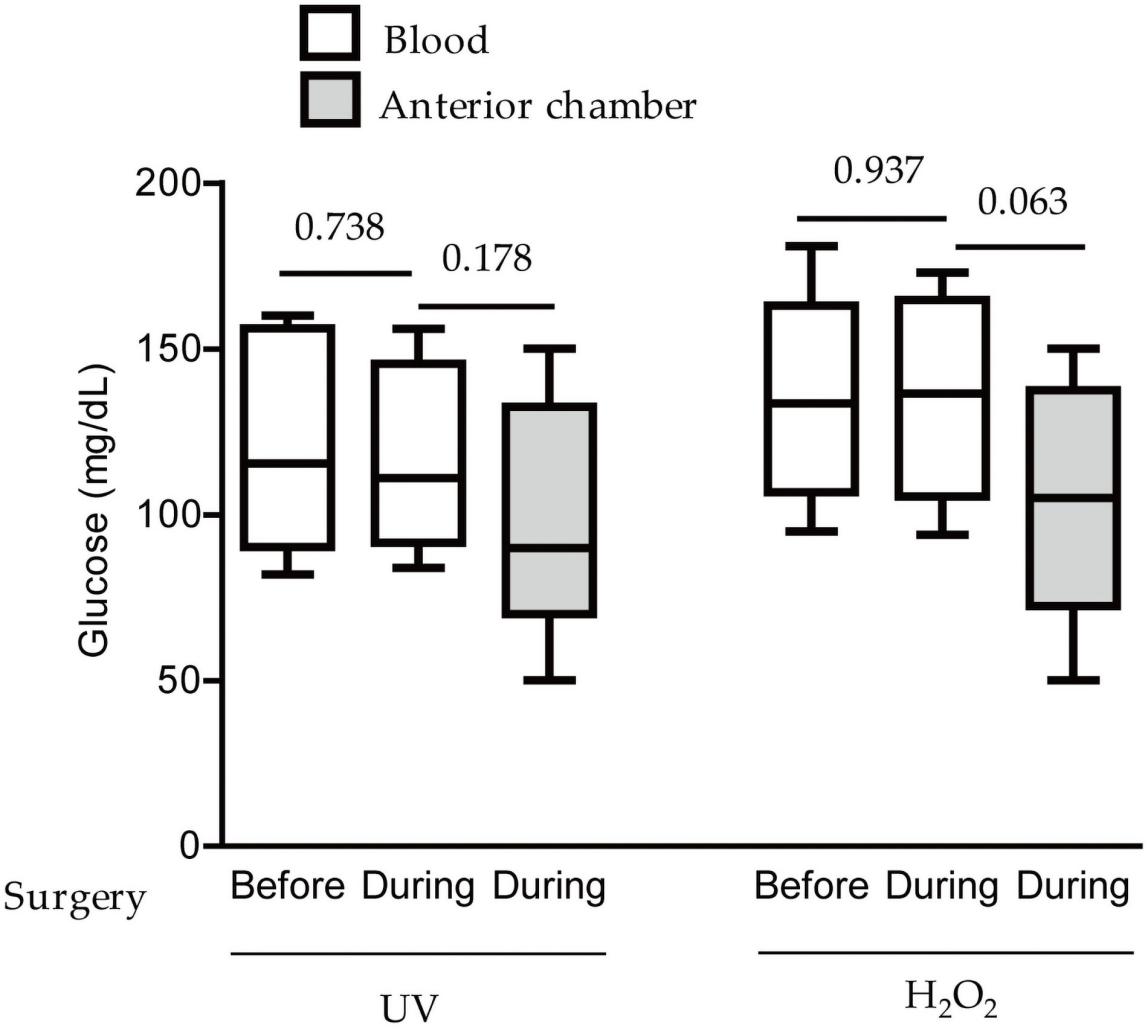
Glucose levels from the anterior chamber and blood in subjects with cataracts in Study 1. Quantitative analyses showed that there was no significant difference in glucose levels between the ‘before/during the surgery’, detected by UV-hexokinase (n = 10) and hydrogen peroxide (H_2_O_2_)-electrode (n = 8; unfortunately, 2 samples were lost during the surgery) methods. In addition, the levels from the anterior chamber and blood were not significantly different, p > 0.05. Graphs are presented as median with interquartile range, the 25th and 75th percentile. The data were analyzed using Student’s *t*-test and one-way ANOVA followed by a Bonferroni post hoc test. UV: UV-hexokinase method; H_2_O_2_: H_2_O_2_-electrode method.

Before the surgery, blood glucose levels of the subjects were evaluated: 118.2 ± 30.90 and 137.0 ± 30.44 mg/dL (mean ± standard deviation), by the two methods (the UV-hexokinase and H_2_O_2_-electrode methods), respectively (Fig 1). Then, blood glucose levels of the subjects were evaluated during the surgery: 117.3 ± 27.62 and 136.9 ± 30.05 mg/dL, by the same methods above (Fig 1). There was no significant difference between blood glucose levels before/during the surgery. While we collected blood samples from the subjects, aqueous humor samples in the anterior chamber were also collected for measuring glucose levels. Glucose levels in the aqueous humor from the anterior chamber were evaluated: 98.0 ± 33.60 and 103.8 ± 35.43 mg/dL by the same methods above (Fig 1). Even though there was no significant difference among glucose levels from the blood before/during the surgery and the levels from the anterior chamber during the surgery, lower values in glucose levels were slightly seen in the aqueous humor than those in the blood (Fig 1).

**Fig 2:**
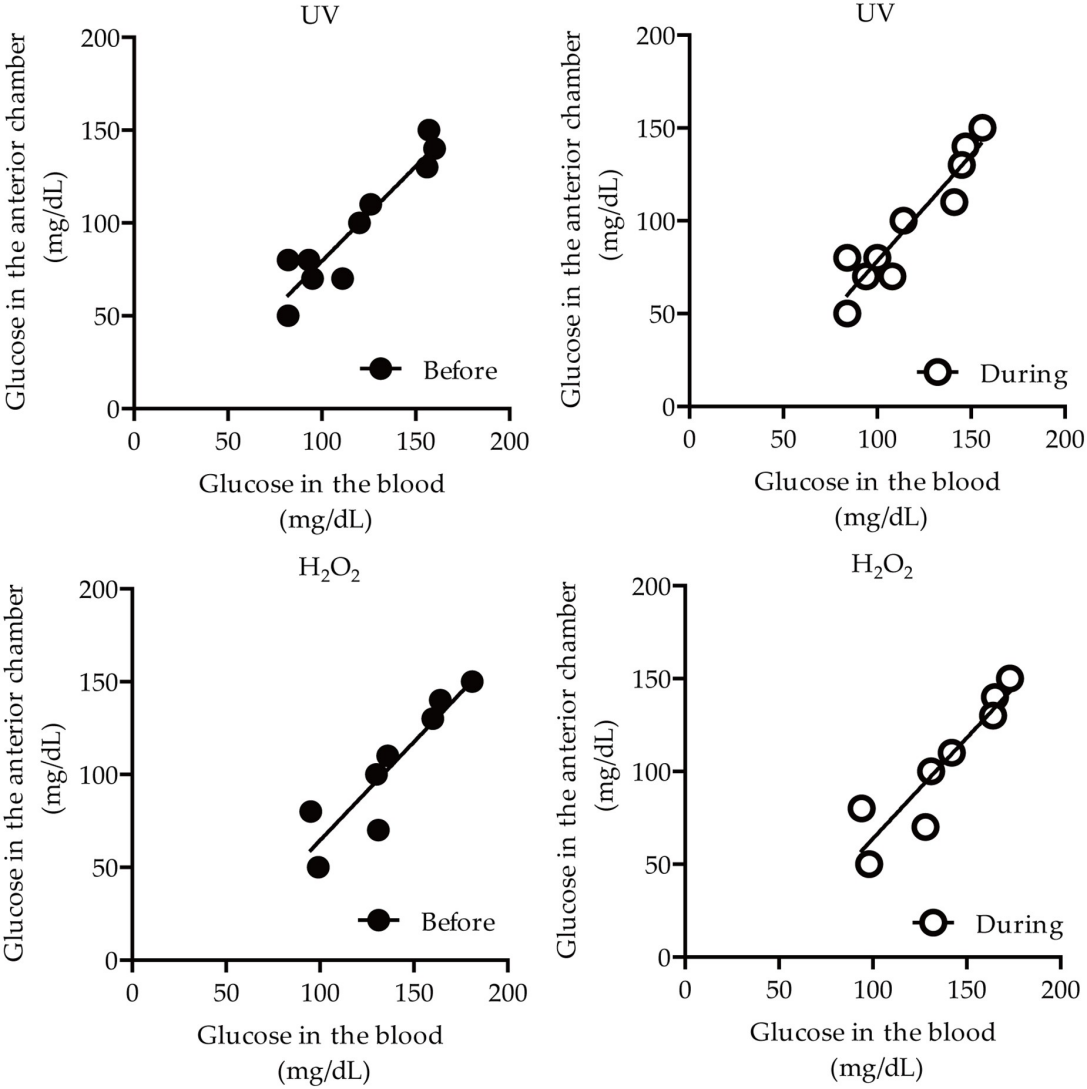
Visualization of a correlation on glucose levels between the anterior chamber and blood in Study 1 subjects with cataracts based on the values in Table 2. Dots represent the measured glucose levels. Lines represent the linear fit of the data points. UV: UV-hexokinase method; H_2_O_2_: H_2_O_2_-electrode method.

Next, correlation analyses on glucose levels between the anterior chamber and blood were performed with the obtained values (Table 2 and Fig 2). Glucose levels from the anterior chamber were significantly correlated with glucose levels from the blood, regardless of the collection time (before/during the surgery) and the methods (UV-hexokinase and H_2_O_2_-electrode methods): R^2^, 0.8672 (before), 0.8678 (during), 0.8019 (before) and 0.8229 (during), respectively (Table 2 and Fig 2).

**Table 2:** A correlation on glucose levels between the anterior chamber and blood in Study 1 subjects.

Measurement/Surgery	Coefficients: Slope (Intercept)	F-statistic	R^2^	95% CI	P-value
UV (n = 10)					
Before	1.021 (-22.709)	59.75	0.8672	0.762–1.280	0.000056
During	1.143 (-36.041)	60.06	0.8678	0.853–1.431	0.000055
H_2_O_2_ (n = 8)					
Before	1.060 (-41.530)	29.34	0.8019	0.676–1.444	0.001638
During	1.086 (-44.881)	33.52	0.8229	0.718–1.453	0.001163

Linear regression analysis (glucose levels from the blood with glucose levels from the anterior chamber). CI: confidence interval. UV: UV-hexokinase method; H_2_O_2_: H_2_O_2_-electrode method.

#### Study 2

Since the correlation lines didn’t cross the origin (x0,y0), which possesses the intercepts (Table 2), we reached two assumptions: (1) simple experimental errors and (2) a concept of a dissociation gap on glucose levels between the anterior chamber and blood. We considered glucose levels in the anterior chamber may not be 100% proportional to blood glucose levels in that it has been reported that glucose levels in the anterior chamber could be lower than glucose levels in the blood and it could be delayed less than 5 mins with complex metabolic mechanisms of glucose dynamics in the body [16, 17]. Due to the time delay, correlation straight lines may not be constant, and it could be estimated that there might be dynamics of glucose levels in the anterior chamber depending on the natural ‘ascending and descending’ periods of blood glucose levels in our body [19]. Thus, we recruited the subjects newly again and started to collect blood samples with aqueous humor samples according to a new experimental scheme (S2 Fig). In this time, only the UV-hexokinase method was used for measuring glucose levels in that we found the better correlation ratios (R^2^ > 0.86) by the UV-hexokinase method, compared with those (0.86 > R^2^ > 0.80) by the H_2_O_2_-electrode method (Table 2).

**Fig 3:**
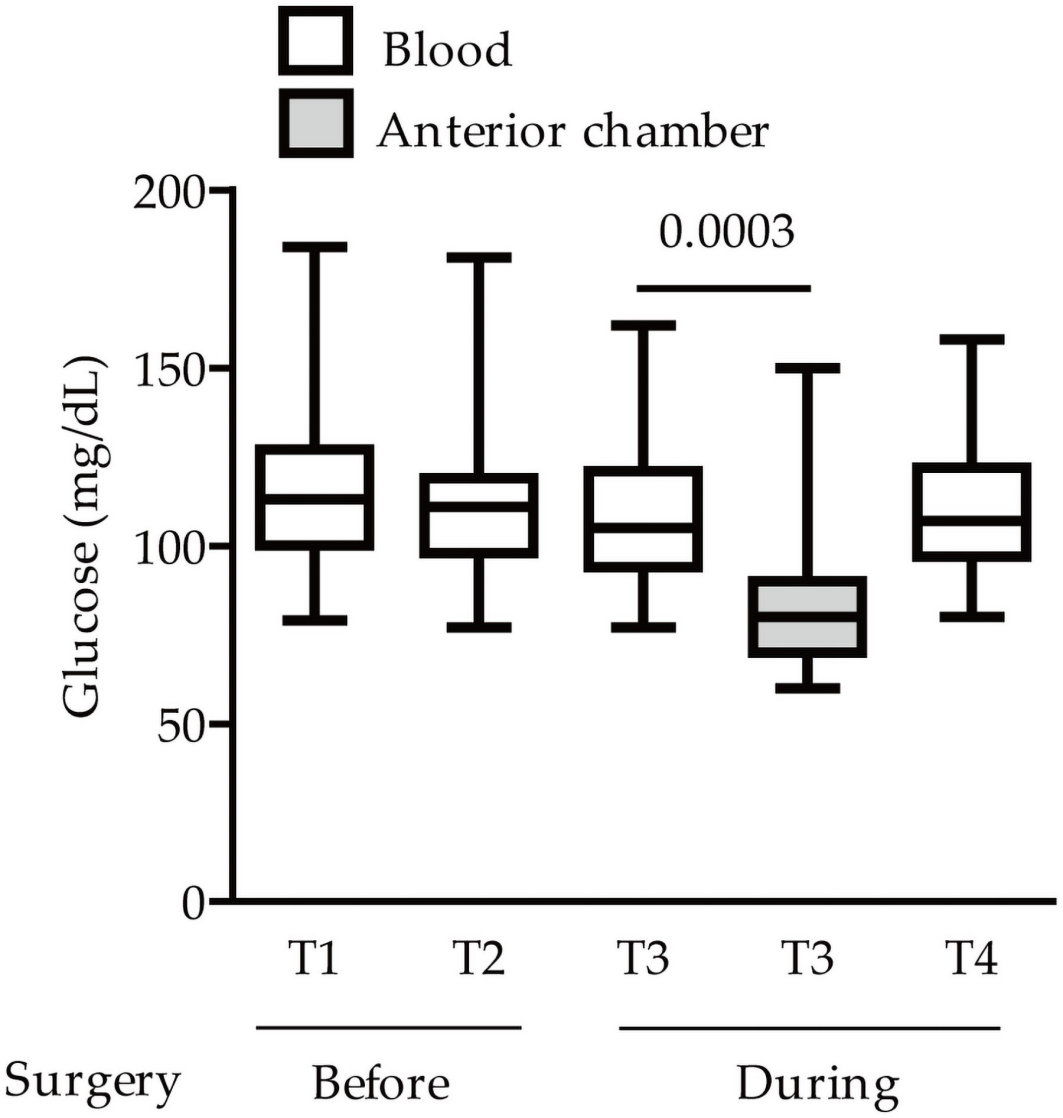
Glucose levels from the anterior chamber and blood in Study 2 subjects with cataracts. Quantitative analyses showed that there was no significant difference in glucose levels between the ‘before/during the surgery’, detected by the UV-hexokinase method (n = 19 from 20; unfortunately, 1 sample had a measurement error during the surgery, hence, it was excluded). The levels from the anterior chamber were significantly lower than those from the blood, p = 0.0003. T1: the time that the patients entered the operation room (before the surgery), T2: after T1 with a 5 mins interval (before the surgery), T3: the timing of surgery, and T4: after T3 with a 5 mins interval (during the surgery). Graphs are presented as median with interquartile range, the 25th and 75th percentile. The data were analyzed using Student’s *t*-test and one-way ANOVA followed by a Bonferroni post hoc test.

First of all, blood glucose levels of the total subjects were evaluated before the surgery: 114.8 ± 22.60 and 112.2 ± 21.45 mg/dL, T1 and T2, respectively (Fig 3). Then, blood glucose levels of the subjects were evaluated during the surgery: 108.2 ± 20.08 and 110.1 ± 18.56 mg/dL, T3 and T4, respectively (Fig 3). At the T3 time point, glucose levels from the anterior chamber were evaluated: 81.05 ± 21.58 mg/dL. While there was no significant difference in blood glucose levels among the groups, glucose levels from the anterior chamber were significantly lower than those from the blood (Fig 3).

**Fig 4:**
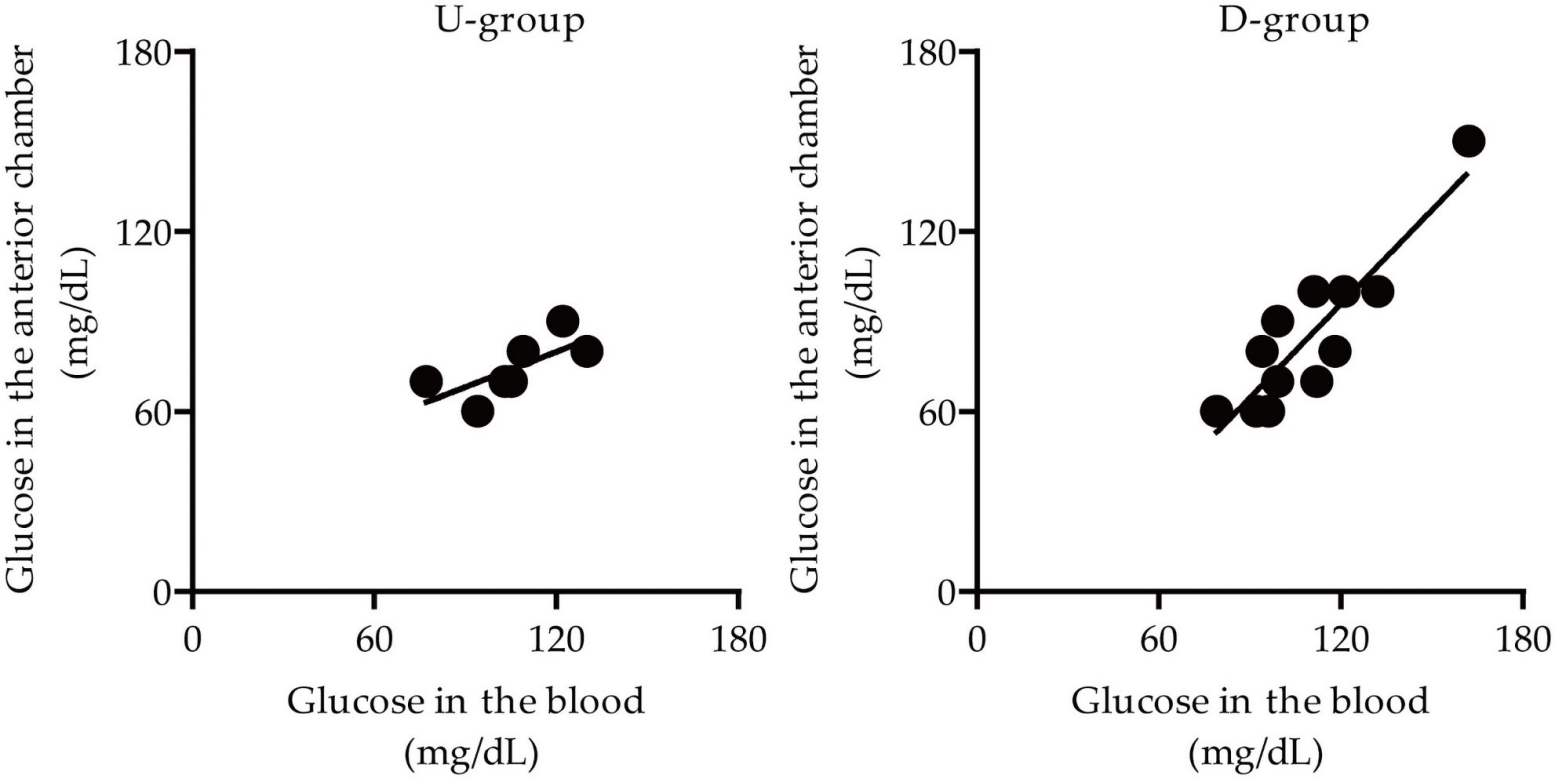
Visualization of a correlation on glucose levels between the anterior chamber and blood in Study 2 subjects with cataracts based on the values in Table 3. Dots represent the measured glucose levels. Lines represent the linear fit of the data points. U: up, D: down.

Next, we divided groups depending on the ‘ascending and descending’ periods of blood glucose levels before/during the surgery: U-group (blood glucose levels-ascending group, Up), which has the calculated value (the average value at the time of T1 and T2 subtracted by the average value at the time of T3 and T4) is above 0, and D-group (blood glucose levels-descending group, Down), vice versa (S3 Fig). Correlation analyses on glucose levels between the anterior chamber and blood were performed depending on these two groups (Table 3 and Fig 4). Glucose levels from the anterior chamber were significantly correlated with glucose levels from the blood in D-group (R^2^ = 0.7777, p = 0.000091) (Table 3 and Fig 4). However, there was no significant correlation on the levels in U-group (R^2^ = 0.3908, p > 0.05) (Table 3 and Fig 4). We also found that there was a (+) intercept value (32.8985) with a low slope coefficient value (0.3915), which has not been seen in the others (Tables 2 and 3).

**Table 3:** A correlation on glucose levels between the anterior chamber and blood in Study 2 subjects.

Measurement/Group	Coefficients: Slope (Intercept)	F-statistic	R^2^	95% CI	P-value
UV					
U-group (n = 7)	0.3915 (32.8985)	4.849	0.3908	0.043–0.739	0.078900
D-group (n = 12)	1.0430 (-29.2920)	39.49	0.7777	0.717–1.368	0.000091

Linear regression analysis (glucose levels from the blood with glucose levels from the anterior chamber). CI: confidence interval. U: up, D: down.

#### Combined results from Study 1 and 2

**Fig 5:**
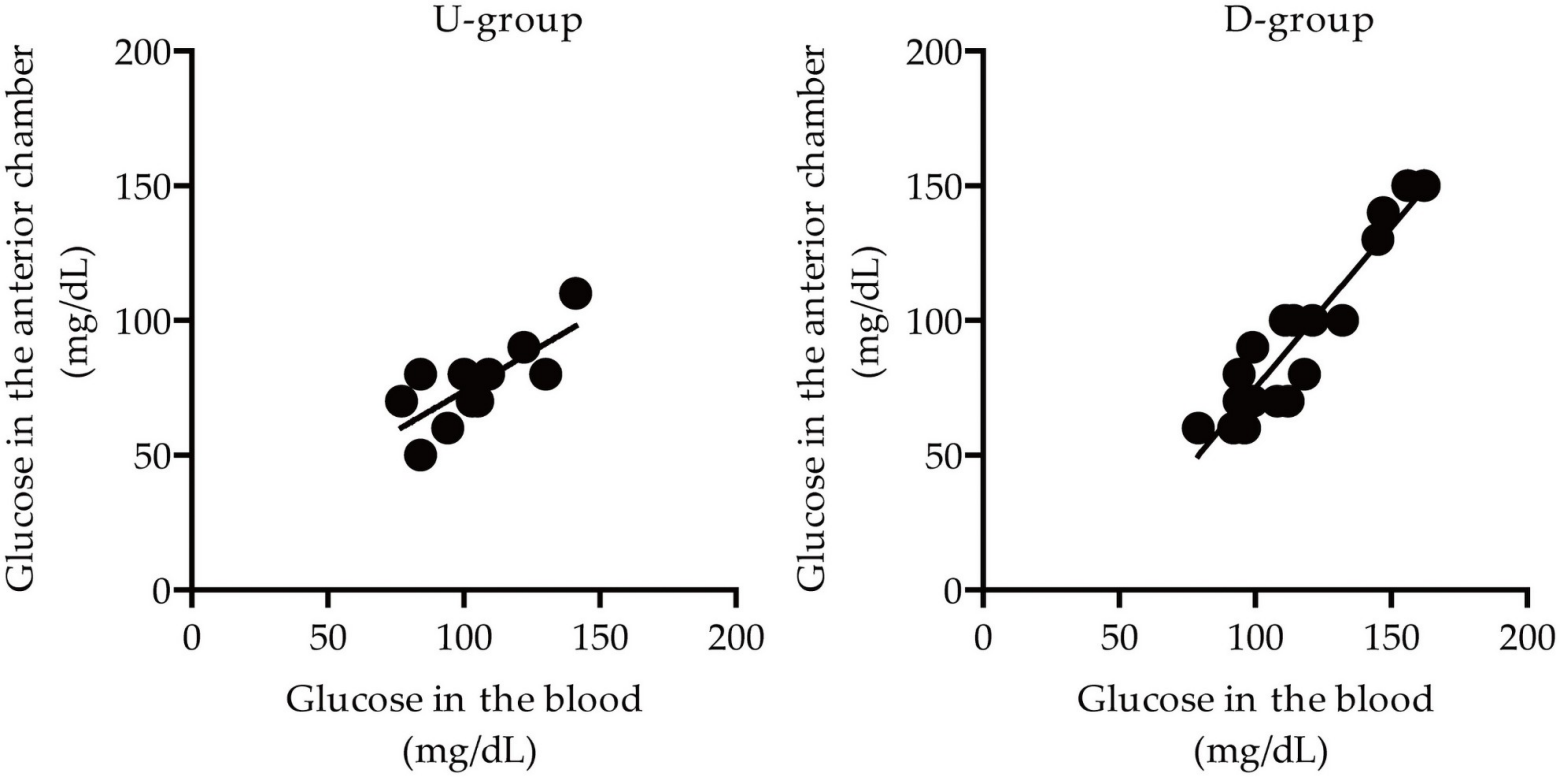
Visualization of a correlation on glucose levels between the anterior chamber and blood in Study 1 and 2 subjects with cataracts based on the values in Table 4. Dots represent the measured glucose levels. Lines represent the linear fit of the data points. U: up, D: down.

At last, we combined Study 1 and Study 2 into one for a final evaluation of the correlation on glucose levels between the anterior chamber and blood. Even though only two time points (before/during the surgery) were set in Study 1, we divided Study 1 subjects into U- and D-groups, simply by the same calculating method used in Study 2. Then, the correlation analyses were performed with the values in the newly made U-group (n = 11, 4 from Study 1 and 7 from Study 2) and D-group (n = 18, 6 from study 1 and 12 from Study 2). As expected, glucose levels from the anterior chamber were strongly correlated with glucose levels from the blood in Study 1 and 2 combined D-group with a high R^2^ value (0.8636) (Table 4 and Fig 5). However, we saw a relatively moderate correlation on glucose levels from the anterior chamber with the levels from the blood in Study 1 and 2 combined U-group with a low R^2^ value (0.5228). Throughout the whole studies, we could see the almost consistent and high slope coefficient and R^2^ values in D-groups (Tables 3 and 4), which showed a strong linear relationship. However, there was the low slope coefficient and R^2^ values in U-groups (Tables 3 and 4), which showed a moderate linear relationship.

**Table 4:** A correlation on glucose levels between the anterior chamber and blood in Study 1 and 2 subjects.

Measurement/Group	Coefficients: Slope (Intercept)	F-statistic	R^2^	95% CI	P-value
UV					
U-group (n = 11)	0.5888 (14.8562)	11.96	0.5228	0.255–0.922	0.00718700
D-group (n = 18)	1.1930 (-44.4630)	108.7	0.8636	0.968–1.417	0.00000002

Linear regression analysis (glucose levels from the blood with glucose levels from the anterior chamber). CI: confidence interval. U: up, D: down.

## Discussion

In the current study, we recruited cataract patients in hospital, measured glucose levels from the anterior chamber and blood, and found the strong correlation on glucose levels between them. Moreover, we divided the subjects based on the blood glucose level dynamics before/during the surgery, and obtained the different correlation ratios depending on the blood glucose dynamics, which is a significance of our study.

Previously, several correlation studies on glucose levels between the anterior chamber and blood have been performed in animals and humans from 1960s [20–25]. For animal studies, because of the size of the eyes and depth of the anterior chamber, only rabbits were suggested as a suitable animal model for measuring ocular glucose levels [9, 14]. Because of a lack of experimental availability regarding the animal models and a difficulty of experimental accessibility for human studies, even though studies have been made for a long time, the correlation ratios for ocular glucose levels to systemic glucose levels still vary depending on the papers [20–25]. It could be attributed to the differences in techniques for measuring glucose levels, collection methods, sample storage, and anesthesia. We also faced the same difficulties in that we obtained the different values for blood glucose levels using the two different measurement methods, although there was no significant difference between them. It could be more complicated to consider the notion that variations in blood glucose levels could be reflected in follwoing variations (such as urea, non-protein nitrogen and so on) in the anterior chamber in disease states [26, 27]. Thus, calculating exact glucose levels from the anterior chamber in vivo is still on the road [17, 28]. Although it is quite hard to conclude that our work solves all existing possible matters, we at least found one factor that could cause a discrepancy in the correlation on glucose levels between the anterior chamber and blood: different correlation ratios for glucose levels between the anterior chamber and blood based on the blood glucose dynamics. In other words, when blood glucose levels ascend, the ratio R (ocular glucose levels divided by blood glucose levels) decreases, and the inclination of the correlation straight line could become relatively low (S4 Fig). Conversely, during the period when blood glucose levels descend, ocular glucose levels often rise late or follow the descending stage close to blood glucose levels, which shows the ratio R inclines to increase and the inclination of the correlation straight line could become relatively high (S4 Fig). We suggest our current findings could help understanding the gap between glucose levels in the anterior chamber and blood.

Frequent determination of blood glucose levels in patients such as diabetes is inevitable for proper managements of the diseases [2]. To date, there have been testing methods such as not only SMBG (especially, repetitive lancing and finger bleeding) but also CGM and FGM. They still have several hurdles regarding efficacy and efficiency. Hence, developing effective and noninvasive detection techniques is of high priority. Many researchers have tried to develop noninvasive detection techniques targerting the eyes, especially the anterior chamber in that the aqueous humor in the anterior chamber is easily accessible glucose-containing body fluid [9–11]. Despite scientific achievements in the development of measurement devices and the validation of the anterior chamber as a noninvasive glucose monitoring site, there are still limitations that need to be overcome before active clinical uses [29]. On the top of that, measuring glucose levels from the anterior chamber in human needs to be established for calculating blood glucose levels more correctly. In this regard, we showed exact glucose levels from the anterior chamber and compared them with blood glucose levels. This could contribute to establishing noninvasive glucose level detection techniques through the anterior aqueous in the anterior chamber.

Our present study still has some limitations. The number of the subjects may not be large enough to generalize our findings although we found the strong correlation on glucose levels between the anterior chamber and blood. The values from Study 1 and 2 subjects were intentionally combined because of the small number of the subjects in the entire study, even though their time points for the glucose collection were not completely matched. This is because the factor for blood glucose dynamics was not deeply considered at the time of Study 1. This matter will be solved in our further studies. In addition, age population of the subjects was not deeply considered during the study. A previous study reported that the stronger positive correlation ratio could be seen in older patients [30]. This matter could also be solved when more subjects are recruited for the study. At last, diabetic patients, whose glucose levels need to be highly measured and managed, were not directly recruited and studied in this study. Those factors might limit our finding’s generalization and scientific significance. Thus, those limitations will be taken into account for further studies.

In conclusion, our findings indicate that blood glucose-ascending or descending condition is important for obtaining the correct correlation ratios for glucose levels between the anterior chamber and blood. This concept could be useful for setting up better noninvasive blood glucose monitoring systems. If data regarding this concept are more stacked, we assure that the correct calculation of systemic glucose levels by measuring ocular glucose levels can be possible. This enables proper and prompt managements for hyperglycemia-mediated diseases including diabetic retinoapthy.

## Supporting information

S1 Fig A general schematic of sample collection in the subjects with cataracts during the surgery. After an operating surgeon (dark red) obtained aqueous humor samples (black) from the anterior chamber of the eye in the subjects (gray) with supports of assistant doctors (light blue), the samples were directly delivered to the table (light green) and glucose levels were measured. At the same time of the aqueous humor sample collection, other assistant doctors with nurses obtained blood samples intravenously and the samples were delivered to the table for the measurement.
(TIF)

S2 Fig A scheme of the sample collection and time points in subjects with cataracts for Study 2. Before the cataract surgery with a 5 mins interval, blood samples (T1 and T2) were collected and measured. During the surgery, blood samples (T3 and T4) were collected with a 5 mins interval. At the first blood sample collection (T3), aqueous humor samples were also collected and measured.
(TIF)

S3 Fig Group distribution depending on the ‘ascending and descending’ periods of blood glucose levels before/during the surgery and blood glucose dynamics from T1 to T4. U-group (blood glucose levels-ascending group, Up, n = 7) has the calculated value (the average value at the time of T1 and T2 subtracted by the average value at the time of T3 and T4) is above 0 and D-group (blood glucose levels-descending group, Down, n = 12 from 13; 1 sample, which showed a reduction of the glucose levels to 54 mg/dL at the time of T3, was excluded for next experiments because of its measurement error) has the calculated value is below 0.
(TIF)

S4 Fig The hypothetical relationship of blood glucose dynamics with glucose dynamics in the Anterior Chamber (AC) of the eye. The ratio R equals the value of glucose levels in AC divided by glucose levels in the blood.
(TIF)

S1 Data (XLSX)
