# Supplementary figures and images for: Glucose levels between the anterior chamber of the eye and blood are correlated based on blood glucose dynamics
Source: PLoS One. 2021 Sep 1;16(9):e0256986. doi: 10.1371/journal.pone.0256986 (PMC8409619; doi:10.1371/journal.pone.0256986)

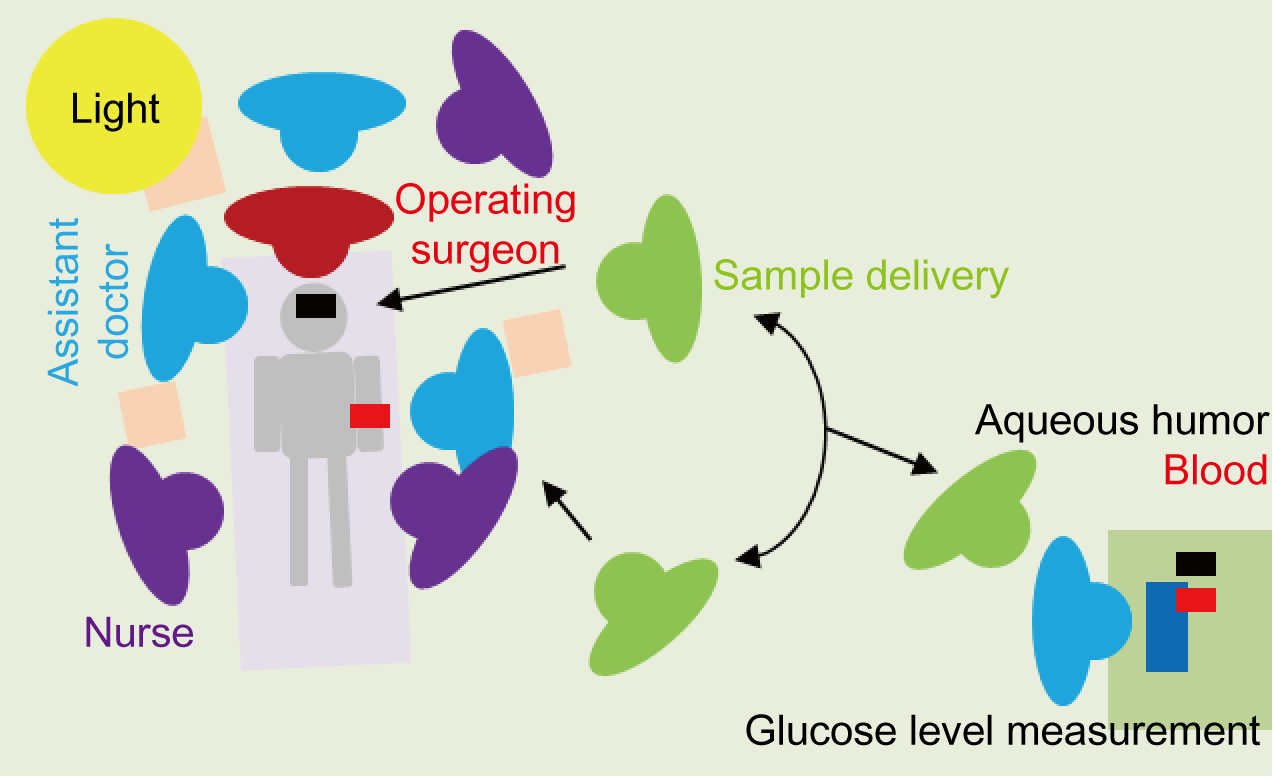

Supplement: S1 Fig — After an operating surgeon (dark red) obtained aqueous humor samples (black) from the anterior chamber of the eye in the subjects (gray) with supports of assistant doctors (light blue), the samples were directly delivered to the table (light green) and glucose levels were measured. At the same time of the aqueous humor sample collection, other assistant doctors with nurses obtained blood samples intravenously and the samples were delivered to the table for the measurement. (TIF) [file pone.0256986.s001.tif]

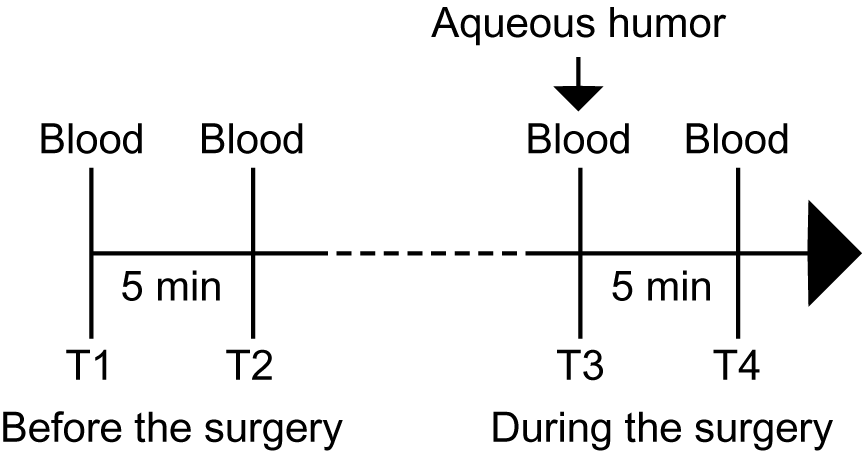

Supplement: S2 Fig — Before the cataract surgery with a 5 mins interval, blood samples (T1 and T2) were collected and measured. During the surgery, blood samples (T3 and T4) were collected with a 5 mins interval. At the first blood sample collection (T3), aqueous humor samples were also collected and measured. (TIF) [file pone.0256986.s002.tif]

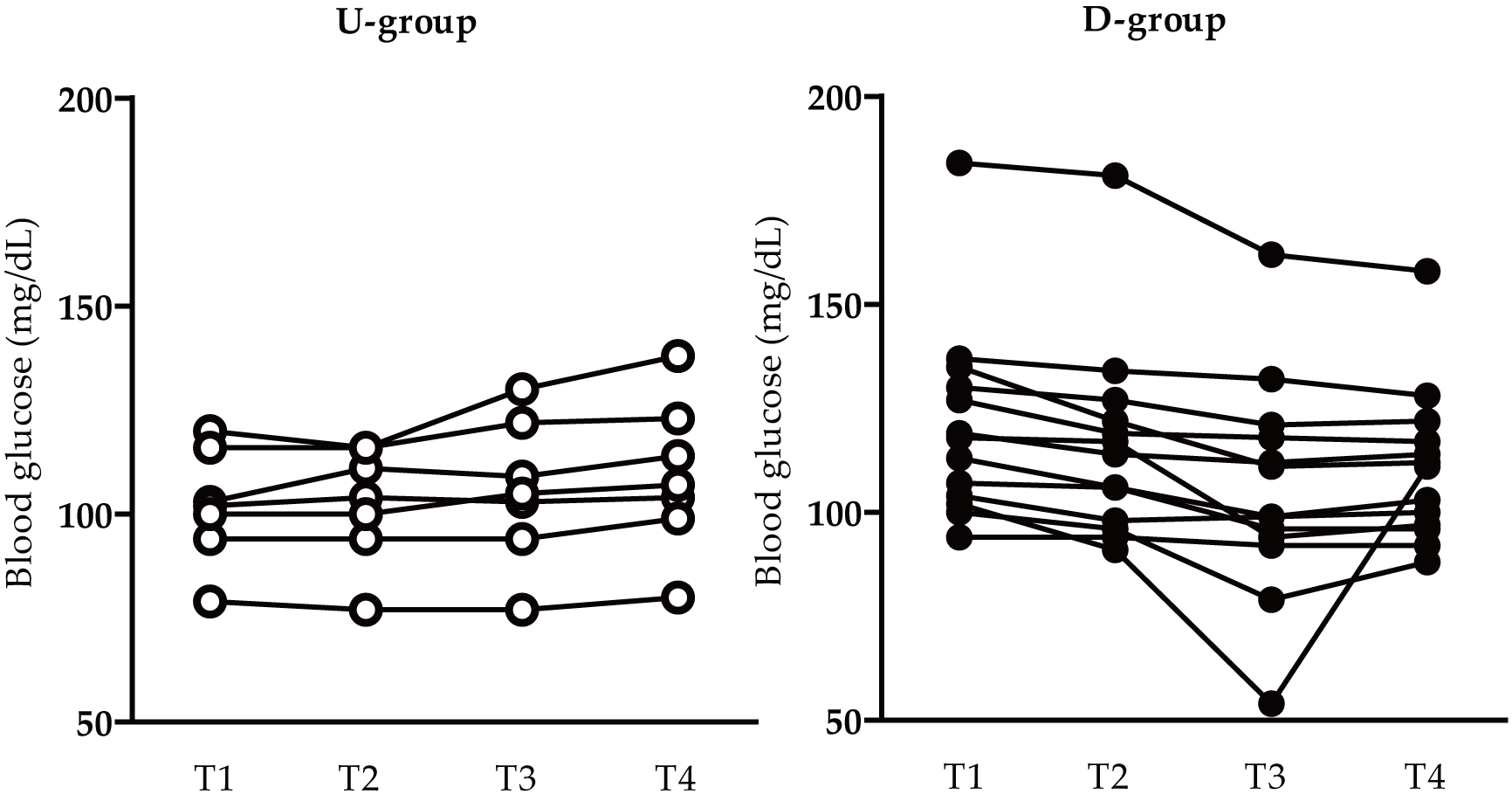

Supplement: S3 Fig — U-group (blood glucose levels-ascending group, Up, n = 7) has the calculated value (the average value at the time of T1 and T2 subtracted by the average value at the time of T3 and T4) is above 0 and D-group (blood glucose levels-descending group, Down, n = 12 from 13; 1 sample, which showed a reduction of the glucose levels to 54 mg/dL at the time of T3, was excluded for next experiments because of its measurement error) has the calculated value is below 0. (TIF) [file pone.0256986.s003.tif]

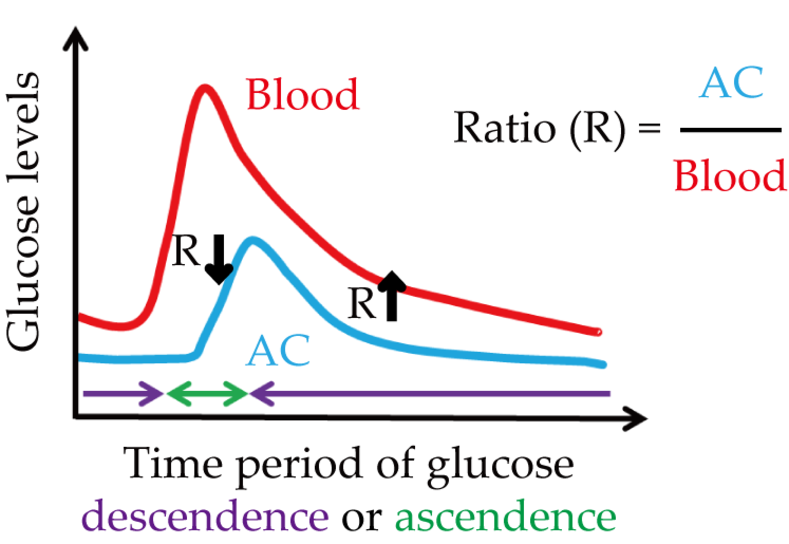

Supplement: S4 Fig — The ratio R equals the value of glucose levels in AC divided by glucose levels in the blood. (TIF) [file pone.0256986.s004.tif]
